# Tire Speckle Interference Bubble Defect Detection Based on Improved Faster RCNN-FPN

**DOI:** 10.3390/s22103907

**Published:** 2022-05-21

**Authors:** Shihao Yang, Dongmei Jiao, Tongkun Wang, Yan He

**Affiliations:** College of Mechanical and Electrical Engineering, Qingdao University of Science and Technology, Qingdao 266061, China; ysh199728@163.com (S.Y.); w1456056728@163.com (T.W.); heyan@qust.edu.cn (Y.H.)

**Keywords:** computer vision, deep learning, object detection, tire defect detection, feature pyramid network

## Abstract

With the development of neural networks, object detection based on deep learning is developing rapidly, and its applications are gradually increasing. In the tire industry, detecting speckle interference bubble defects of tire crown has difficulties such as low image contrast, small object scale, and large internal differences of defects, which affect the detection precision. To solve these problems, we propose a new feature pyramid network based on Faster RCNN-FPN. It can fuse features across levels and directions to improve small object detection and localization, and increase object detection precision. The method has proven its effectiveness through cross-validation experiments. On a tire crown bubble defect dataset, the mAP [0.5:0.95] increased by 2.08% and the AP0.5 increased by 2.4% over the original network. The results show that the improved network significantly improves detecting tire crown bubble defects.

## 1. Introduction

Tires are the only medium for cars to contact the ground. According to the World Health Organization, tires contribute to 40% of all traffic accidents [1]. Therefore, the quality of tires is important for the driving safety of cars and tire quality inspection is critical in the tire production. In the tire industry, the quality inspection of tires in many factories still adopts the quality inspection method of manual visual observation. This method is inefficient, subjective, labor-intensive, and has a high missed inspection rate, which can not meet tire manufacturing’s automation requirements. However, some existing automated defect detection methods are based on manual design, which needs to design algorithms for each defect, which is complex and not robust [2,3]. In recent years, automatic inspection based on deep learning has been developed for industrial inspection applications such as steel [4], fabrics [5], solar batteries [6], etc. Many scholars have applied neural networks to traffic cars [7,8,9]. Specific to tire production scenarios, there are more and more tire defect detection methods based on deep learning [10,11,12]. This detection technique is more effective than manual detection. Compared with traditional visual inspection techniques, it needs less prior knowledge of designers and does not need to design inspection algorithms for each defect.

Since 2012, after the team led by Hilton proposed the convolutional neural network (CNN)—Alexnet—and won the imageNet (large scale visual recognition challenge championship), the object detection based on neural network has developed rapidly. In [13], the authors propose the faster regions with CNN features (Faster RCNN), which is a high-precision two-stage detection network. The authors propose the region proposal network (RPN), which can use a convolutional neural network to make candidate regions to distinguish foreground and background, replacing the traditional Selective Search [14]. It speeds up the detection speed and precision. In [15], the authors propose the feature pyramid network (FPN). FPN can fuse the high resolution of low-level features and high semantic information of high-level features output from the backbone network to improve object detection. Since then, more scholars have continued to explore FPN. In [16], the authors propose PAFPN, which add a bottom-up secondary fusion network to the FPN. Based on FPN, PAFPN adds a bottom-up fusion path to improve the entire feature hierarchy, making the underlying positioning signal accurate and shortening the information path between the lower and uppermost features. It improves the effect of object detection and region segmentation. In COCO 2017, PAFPN won the champion of instance segmentation and the second place of object detection. In [17], the authors propose a balanced feature pyramid (BFP), it scales the feature map to a uniform size and accumulates the average, and then refines the averaged features through a non-local neural network. Then, the fused feature maps are for four feature maps with the same size as the original feature layer, and then added to the feature maps of the original feature layer to achieve the effect of feature improvement. In [18], in the FPN of the Yolov3 network, the authors combine the feature maps of each stage again with the feature maps of the other three stages, and use attention to control the weight of feature map fusion for fusion features at different stages in FPN. This method achieves a balance between speed and precision. In [19], Google adopts the reinforcement learning method to search the NAS-FPN on the RetinaNet network. It shows strong object detection performance on Imgnet, but needs to spend time on many of TPUs to find the best architecture. In [20], the authors propose a feature pyramid grid (FPG), which fuses the feature maps horizontally and vertically multiple times to form a unified feature pyramid grid. It has higher precision than the FPN on the detection and segmentation. The above network has improved the effect of object detection, but it is only on the COCO dataset or the Image dataset, and it is not specific to a certain industrial scene. When the neural network is applied to a specific industrial scene, it should be optimized for this scene.

## 2. Background and Related Work

In the tire industry, as a rubber product, tires are composed of composite materials. There are many types of tire defects, and bubbles are one of the most common quality defects in tires. The location where the tire crown is prone to bubbles is mainly between the tread and the belt layer, between different belt layers, between the belt layer and the carcass, the end of the belt layer, and the joint between the belt layer and the sidewall. It has a great potential harm to the safety performance of tires, and it is easy to cause tire shoulders, delamination, and even puncture. Therefore, tire bubble defect detection is critical in the tire production.

Because the COCO or Image dataset is different from the tire defect dataset detection object, the COCO or Image dataset includes things common in nature, such as people, cars, cats, dogs, etc. However, a tire defect dataset includes grayscale pictures, which have the characteristics of similar background to the object, low contrast, and small object size. Therefore, tire bubble defect detection cannot directly use classical neural networks that perform well on COCO or Image datasets. It is necessary to adapt and improve the neural network according to the characteristics of the object to make it better in detecting tire crown bubble defects. For the bubble defect detection of tire crown, there are problems when object and background are aliased and difficult to distinguish, and the small size of the detected object affects the detection effect. Therefore, the neural network needs to adjust to solve these difficulties and make it better for tire production. It is important to improve the safety of tires.

Over the past few years, many researchers have applied neural networks to nondestructive testing of tires. In [10], the authors propose a tire defect detection method based on a concise semantic segmentation network. They propose segmentation networks and compact convolutional neural networks for tire defect detection, resulting in smaller model size and faster detection. In [11], the authors propose a tire image defect detection method based on a fully convolutional neural network. They replaced the fully connected layer with a convolutional layer in Vgg16 and upsampled and summed each feature map. Finally, it produces an output of the same size as the original image, which is used to locate and segment defects in the image. In [21], the authors introduce a variable convolutional neural network into the Faster RCNN, adopt a multi-scale RPN, and use background features to reorder the candidate boxes to improve tire detection’s precision. In [22], the authors propose an algorithm for tire defect detection and classification based on the RPN. For the problem of large span of tire defect scales, they use the different layers of the convolutional neural network to hierarchically design defects of different scales. This method improves the effect of tire object defect detection.

The above neural networks have improved the effect of tire defect detection, but there are still some problems. First, most researchers study tire X-ray defect images, and there are fewer studies on speckle interference tire defect images. Speckle interference tire defect images have lower contrast, and the object is more similar to the background. Second, some networks include image segmentation. For industrial production, pixel-level segmentation of defects is not needed but only precision detection of defects. This module increases the complexity and computation of the network, which is not conducive to the actual network model deployment.

In this paper, based on the difficulty of detecting tire crown bubble defects, we design a multi-directional integration feature pyramid network called tyre-FPN (TY-FPN). Cross-validation on the tire crown bubble defect dataset with mAP (mean average precision) [0.5:0.95] and AP (average precision) 0.5 as evaluation metrics. AP0.5 means that, when the intersection over union (IOU) of the prediction box and the ground-truth box is greater than 0.5, the prediction box recorded as correct detection; under this condition, the average precision was obtained for all detected images. In addition, mAP [0.5:0.95] takes 10 values of AP’s IOU from 0.5 to 0.95 every 0.05 to calculate the mean AP. The higher the mAP [0.5:0.95] and AP0.5, the higher the detection precision and the lower the missed detection rate. At the same time, the higher the mAP [0.5:0.95], the bigger the precision defect’s location and size. The experimental results show that: mAP [0.5:0.95] and AP0.5 increase by 2.08% and 2.4% respectively. The detection effect of TYFPN is significantly better than that of FPN.

## 3. Improved Network Algorithm Based on Faster RCNN-FPN

Our work built on Faster RCNN-FPN, and we improved FPN to make it better for tire bubble defect detection scenarios.

### 3.1. Detection Process and Image Characteristics of Tire Crown Speckle Interference Bubble Defect Image

**Figure 1:**
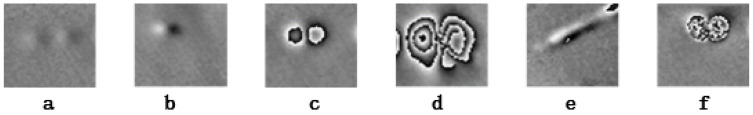
Tire Speckle Interference Bubbles. (**a**–**f**) are the bubble part of the tire laser speckle interference bubble defect image, the pixel size of these images is 67 × 67, and the size of the bubble defect is lower than 32 × 32, which belongs to the size range of small objects.

Speckle interferometry is to use coherent light to brighten the surface of a rough object, and the speckle formed in space can detect the displacement and deformation of the object surface. This technology has been widely used in tire crown bubble defect detection [23]. The speckle interference tire bubble defect detection is as follows: First, all the detections are installed in a room to apply different pressures to the tire to perform segmental detection. Then, the surface irradiated by coherent light is recorded with a CCD camera and the image to the computer. Finally, a computer preprocesses the image and detects a bubble defect. Figure 1 is a tire crown speckle interference bubble defect image. The size of each image is only 67 × 67, and the proportion of bubbles in the image is less than 50%, and the size of the bubbles is less than 32 × 32, which belongs to the small object detection range. From Figure 1, we can intuitively see the characteristics of the tire speckle interferometry bubble:

1. Tire speckle interference images have low contrast and low brightness;
2. Tire crown bubble defects vary widely: There are many styles of bubble defects;
3. As shown in Figure 1a, the bubble defects are very similar to the background and are difficult to distinguish with the naked eye compared with Figure 1b,c;
4. As shown in Figure 1f, the bubble defects are fully manifested and the uncorrelated effect obviously destroys the conditions of speckle interference, making the phase values appear chaotic fringes;
5. On the whole, tire bubble defects are often small objects, and the small scale of small objects makes the feature pixels easy to weaken or even disappear after multiple pooling in the neural network.

### 3.2. Improved Network Algorithm

FPN fuses high-level feature map information from top to bottom to low-level feature maps and builds a feature pyramid network. It obtains more feature information and outputs in different feature layers, improving object detection performance. However, when it is directly applied to tire crown bubble defects’ detection, it needs to adjust according to the characteristics of the bubble defects and the difficulty of bubble defect detection. It can not be directly applied to tire bubble defect detection, and the network still has room for improvement:

1. The high-level feature map of FPN only adds and fuses the adjacent feature maps downward, without upward or cross-scale fusion. Therefore, the high-level feature map does not make full use of the location information of small objects in the low-level feature map. There is still room for improvement in small objects’ detection;
2. For the tire crown bubble defect dataset, the defects belong to the detection range of small objects, and we do not need to consider large objects’ detection. Therefore, more low-level feature map information can be fused into high-level feature maps.

**Figure 2:**
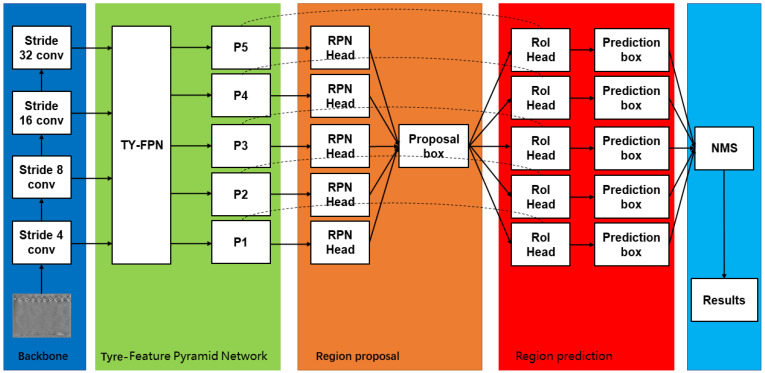
Improved bubble defect detection algorithm. It includes four parts: Backbone, Tyre-Feature Pyramid Network, Region proposal, and Region prediction.

Based on the above ideas, we designed a multi-directional fusion feature pyramid network named TYFPN. It not only fuses the semantic information of the high-level feature map into the low-level feature map, but also fuses the location information of the low-level feature map into the high-level features. This can make a high-level feature map improve the effect of detecting small objects to improve the overall detection effect. There is an improved bubble defect detection algorithm shown in Figure 2. The backbone network is ResNet50 [24]. The backbone network outputs four feature maps of different sizes, and then the feature maps are into TYFPN for feature fusion and feature improvement. TYFPN will output five feature maps, which will enter the RPN network for regional proposal operations, produce many proposal boxes, and then obtain the prediction boxes in RoiHead, filter the detection results through non-maximum suppression (NMS), and finally pass the loss function to calculate the loss.

### 3.3. TYFPN: Multi-Directional Fusion Feature Pyramid Network

**Figure 3:**
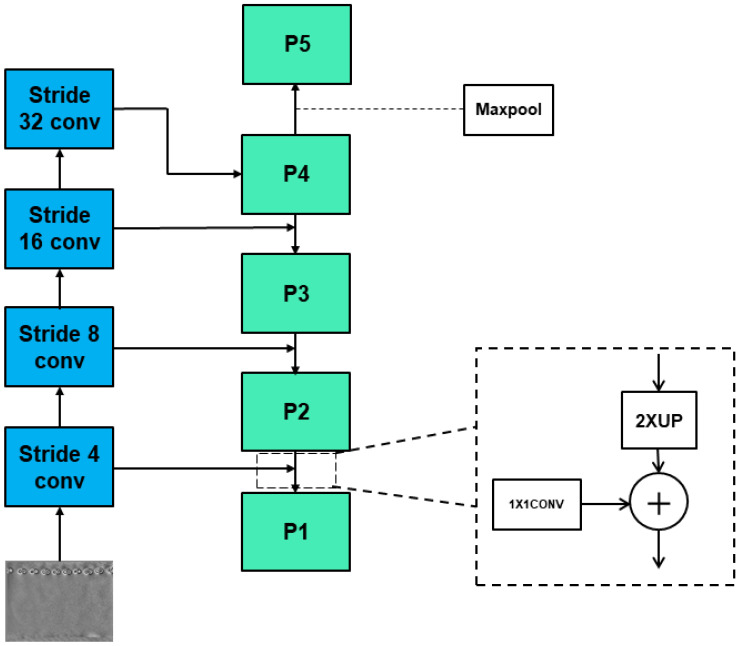
Feature Pyramid Network—including a bottom-up feature extraction, a top-down feature fusion, and lateral connections.

The FPN shown in Figure 3. It performs 1 × 1 convolution on the four feature maps output by ResNet50, and then performs feature fusion from top to bottom. When high-level feature maps are fused with low-level feature maps, the rich semantic information of high-level feature maps is fused into low-level feature maps, so low-level feature maps obtain more useful information about objects.

The high-level feature map of FPN is effective for detecting large objects, but the bubble defects in the tire crown are often small objects. In addition, FPN only has top-down feature fusion of adjacent feature maps, low-level feature maps obtain semantic information of high-level feature maps, but high-level feature maps do not obtain location information of low-level feature maps. Therefore, there is still some room for improvement in high-level feature map detection of objects. In addition, FPN pays more attention to the feature fusion of adjacent layers. When there is a certain span between the low-level feature map and the high-level feature map fusion, the location information is not necessarily accurate, and its features will be weakened during the fusion [25].

**Figure 4:**
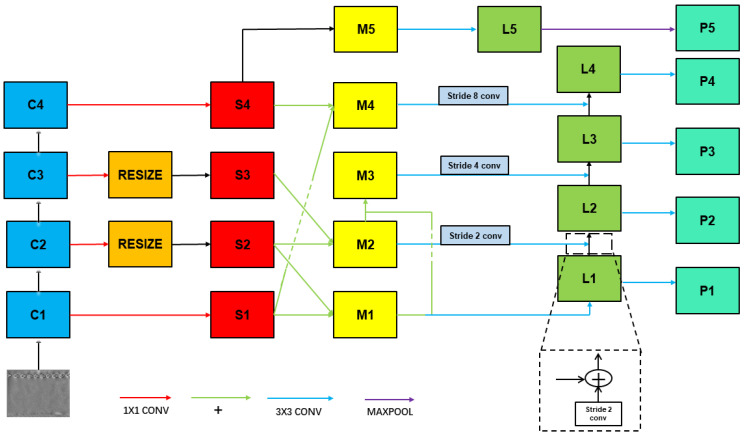
Tyre—Feature Pyramid Network—including a bottom-up feature extraction, a multi-directional, cross-level feature fusion, and lateral connections.

Based on the above ideas, we fuse the rich location information of the low-level feature map into the high-level feature map to make the positioning more accurate and improve the detection effect of small objects. The improved feature pyramid network shown in Figure 4. Firstly, ResNet50 outputs [C1 C2 C3 C4] four feature maps for 1 × 1 convolution, and the number of channels is uniformly 256. Then, the [C2 C3] feature layer is upsampled with nearest neighbor interpolation to make [C2 C3] have the same resolution as a [C1] feature layer, and the output feature layers are [S1 S2 S3 S4]. The nearest neighbor interpolation method is as Equation (1):

(1) F(X,Y)=F(W/w∗x,H/h∗y)

Among them, W, *w*, H, and *h* are, respectively, the width after enlargement, the width before enlargement, the length after enlargement, and the length before enlargement. The pixel value of the enlarged image pixel point (*x y*) corresponds to the pixel value of the original image pixel point (W/*w* ∗ *x* H/*h* ∗ *y*) (rounded off for decimals). Secondly, the [S1] feature layer adds an [S2] feature layer to become the [M1] feature layer. The [S2] feature layer adds an [S3] feature layer to become an [M2] feature layer. The [M1] feature layer adds an [M2] feature layer results in the [M3] feature layer. The [S4] feature layer is added to the [S1] feature layer to become the [M4] feature layer, and the [S4] feature layer becomes the [M5] feature layer. [M1 M2 M3 M4 M5] feature layer for 3 × 3 convolution, [M2 M3 M4] feature layer downsample, [M2 M3 M4] feature layer’s size becomes 1/2, 1/4, 1/8 of themselves. After processing, [M1 M2 M3 M4 M5] feature layer becomes the [L1 L2 L3 L4 L5] feature layer. Finally, the [L1 L2 L3 L4] feature layer up-sample from bottom to top and add to the adjacent layers, max pooling on the [L5] feature layer. This obtains the [P1 P2 P3 P4 P5] feature layer and processes it in the next module.

TYFPN not only has feature fusion between adjacent layers, but also fusion between spanning layers. There is not only the fusion of high feature maps to low level feature maps, but also the fusion of low feature maps to high level feature maps. It makes full use of the small object information in the lowest feature layer. In the tire crown bubble defect dataset, TYFPN performs better than FPN, and tire crown bubble defects’ detection effect has been significantly improved. The experimental and results will be described in detail in Section 3.

### 3.4. Anchor Box Setting and Sample Balance

**Figure 5:**
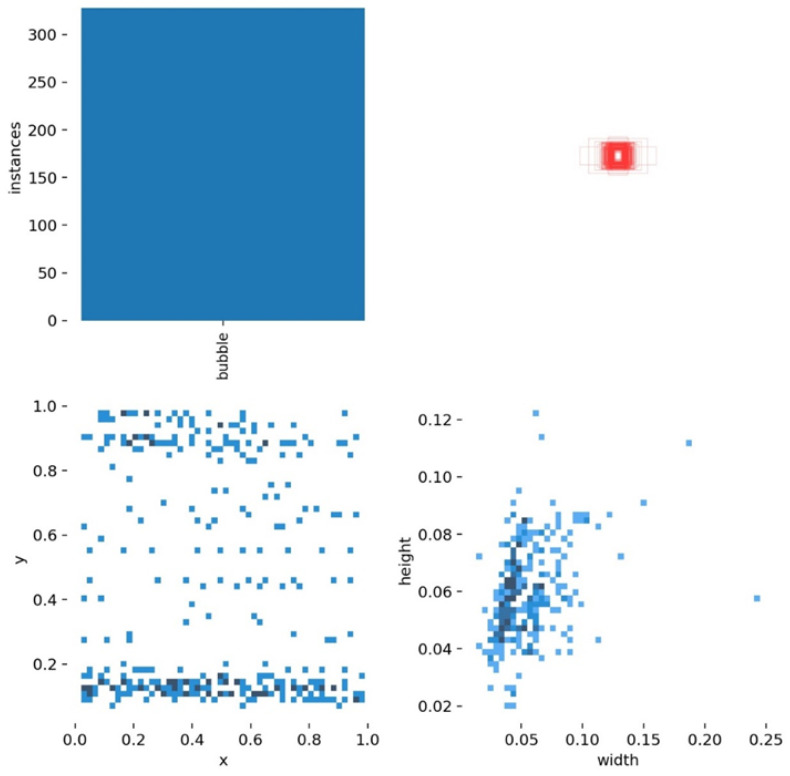
Bounding box coordinates and size distribution plot. Statistical image of the number of bubble defects (**upper left**); All bounding box size images (**top right**); Bounding box relative position distribution image, *x* and *y* are the coordinates of the relative position of the bubble in the image. (**bottom left**); the height and width of the bubble (**bottom right**).

In Faster RCNN, the anchor box provides region proposals for the ROI (region of interest), and the size and aspect ratio of the anchor box have a great influence on the detection. The setting of the anchor box needs to be based on the distribution of the size and aspect ratio of the objects in the dataset. We performed an analysis on the tire crown bubble defect dataset and the results shown in Figure 5. From Figure 5, the size of the object is very small, and the aspect ratio of the ground truth frame of the object is basically distributed around 1, but there are also a small number of objects of other ratios. Therefore, we set the base size of the anchor box to 8 and its aspect ratio to 0.5, 1, 2. For many anchor boxes made by RPN, we process them as follows:

Step 1 Set each anchor’s mask to −1, indicating that the anchor is neither a positive sample (objects) nor a negative sample (background);
Step 2 Set anchors’ mask to 0, if maximum IoU with all ground-truths is less than 0.3, indicating negative samples;
Step 3 Set anchors’ mask to 1, if max IoU with all ground-truths more than 0.7, indicating a positive sample;
Step 4 Some ground-truths are not assigned to find the anchor with the largest IoU. If the IoU is greater than 0.3, set this anchor as a positive sample;
Step 5 Limit the number of training samples, balance positive and negative samples, and set the ratio of positive and negative samples to 1:1, for a total of 256 samples;
Step 6 If the number of positive samples is less than 128, they are filled with negative samples.

### 3.5. Loss Function

As shown in Equation (2), the loss includes the RPN loss and the ROI loss, and the RPN loss and the ROI loss each include the classification loss and the bounding box loss. The same loss function is used for the classification loss and the bounding box loss for the RPN and ROI. In addition, the classification loss is the cross-entropy loss function as Equation (3) and the bounding box loss is the l1 loss function as Equation (4). In Equation (3), $p_{i}$ represents the probability that the *i*-th anchor box is predicted to be the true label; when the positive sample *y* is 1, the negative sample *y* is 0. In Equation (4), $y_{i}$ is the predicted value, ${\hat{y}}_{i}$ is the true value, and $|y_{i}-{\hat{y}}_{i}|$ is the absolute value of the difference between the predicted value and the true value:

(2) $$Loss=Loss_{rpn}+Loss_{roi}=Loss\_rpn_{cls}+Loss\_rpn_{bbox}+Loss\_roi_{cls}+Loss\_roi_{bbox}$$

(3) $$Loss\_rpn_{cls},Loss\_roi_{cls}=-\sum_{i=1}^{k}\left[y^{*}\log\left(p_{i}\right)+\left(1-y^{*}\right)\log\left(1-p_{i}\right)\right]$$

(4) $$Loss\_rpn_{bbox},Loss\_roi_{bbox}=\sum_{i=1}^{k}\left|y_{i}-{\hat{y}}_{i}\right|$$

## 4. Experiment and Analysis

### 4.1. Experimental Setup

**Figure 6:**
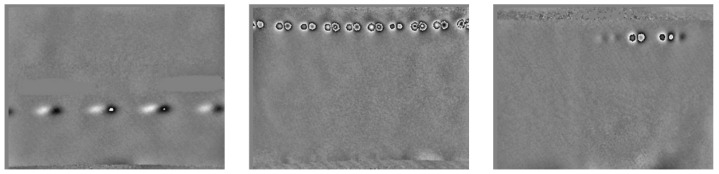
Speckle interference tire bubble defects example. These are some instances in the dataset, and the places with higher gray values in the image are bubbles.

The dataset is the tire crown bubble defect images obtained by the phase shearing speckle interference technology, including 463 bubble defect samples. The ratio of the training and test datasets is 7:3. To prevent overfitting, the images are stretched, scaled, and randomly radially transformed for data enhancement. There are some examples from the dataset in Figure 6.

**Figure 7:**
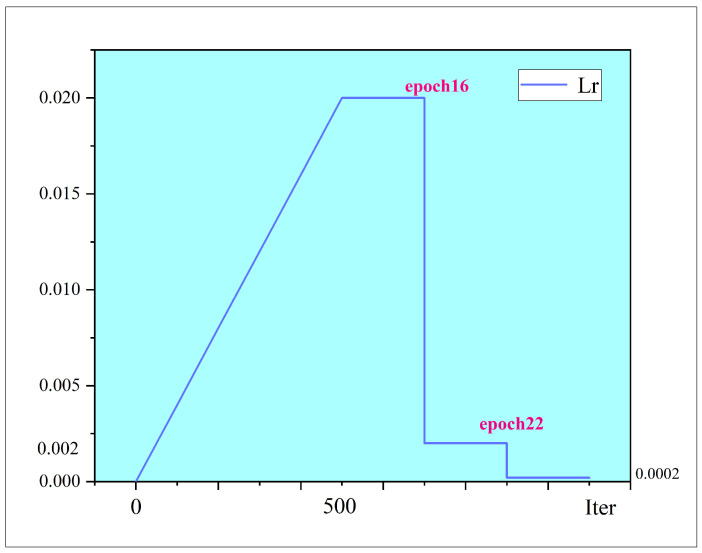
Learning rate settings. The learning rate increases linearly to 0.02 for the first 500 iterations and decreases to 10% at epoch 16 and epoch 22.

The settings of some parameters and details of the training shown in Table 1, 24 training epochs are set, and the learning rate is set as shown in Figure 7. During the first 500 iterations, the learning rate increases linearly to 0.02, and at epochs 16 and 22, the learning rate decreases to 10%.

**Figure 8:**
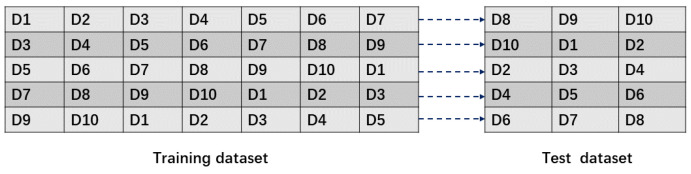
Schematic diagram of the cross-validation experiment. The ratio of training set to test set is 7:3, and k-fold cross-validation is performed, k = 5.

We use cross-validation to ensure that each image is used for training and testing, and the cross-validation method is shown in Figure 8. The ratio of training and test sets is 7:3, and the dataset is considered as consisting of 10 copies of the same number of images. Each time, seven copies are used for training and three copies for testing, and the process is repeated 5 times. As in Equation (5), the result of the ith experiment is recorded as $E_{i}$, and its average value is taken as the final evaluation metric $\bar{E}$.

(5) $$\bar{E}=\frac{1}{n}\sum_{i=1}^{n}E_{i}$$

### 4.2. Bubble Defect Detection Results with Different Algorithms Based on FPN

We compare TYFPN with some state of the art (SOTA) methods on mAP [0.5:0.95] and AP0.5. As evaluation metrics for detection tasks, the higher the mAP [0.5:0.95] and AP0.5 values, the better the performance of the algorithm. The experimental results are shown in Table 2 and Table 3. Table 2 is the result of taking mAP [0.5:0.95] as the evaluation index, and Table 3 is the result of taking AP0.5 as the evaluation index.

**Figure 9:**
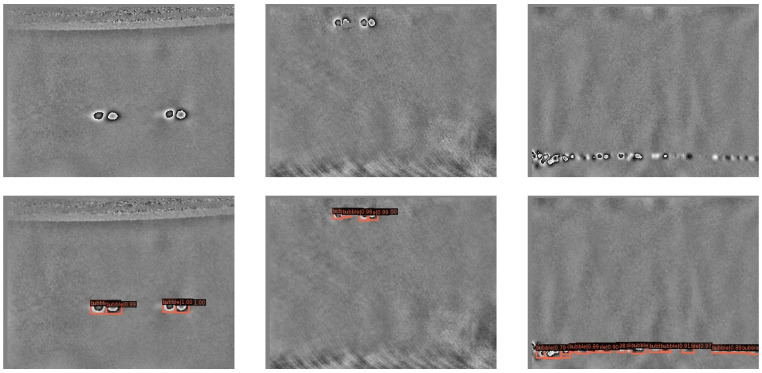
Tire bubble defect images and detection results. The upper part is the picture with defects, and the lower part is the corresponding detection result, including the predicted defect location, boundary, and probability.

The average value of the five experimental results is calculated by Equation (5), and the results are shown in Table 4. It can be seen from Table 4 that the improved network has significantly improved the detection effect of the tire crown bubble defect dataset compared with Faster RCNN-FPN, in which mAP [0.5:0.95] reaches 51.86%, an increase of 2.08%, and AP0.5 reached 93.78%, an increase of 2.4%. Compared with the detection performance of other different algorithms based on FPN, it also has a better detection effect. Some of the detection results of our algorithm are shown in Figure 9. The upper image in Figure 9 is a speckle interference image of a tire with defects, and the lower image is the resulting image of defect detection by our network model. We can find that more than 95% of bubble defects can be detected by our method. In Figure 9, the red detection box represents the position of the bubble defect, the boundary of the detection box represents the size of the bubble defect, and the number represents how confident the computer is that it is a bubble defect. With this information, we can know the size, location distribution of bubbles, and samples of difficult-to-detect bubbles. In this regard, it can be further inferred which processes have problems, which positions and which types of bubbles are difficult to detect, so as to further adjust the production technology and improve the detection method.

### 4.3. Ablation Studies

The above experimental results show that Faster RCNN-TYFPN is better than other networks for tire crown bubble defect detection, but different network structures have differences in many aspects, such as backbone, neck, loss function, and so on. To further demonstrate that the improved network boosting effect is caused by our proposed TYFPN, we design an ablation experiment. Under the same conditions except the neck of the network, the detection results of using TYFPN and not using TYFPN are compared, and various SOTA backbones are replaced at the same time to observe the effect. The detection results of using TYFPN and not using TYFPN under different backbones are shown in Table 5: for different backbones, when TYFPN is used, the detection effect is improved to varying degrees. Under the condition of using TYFPN as the neck, when ResNet50 is the backbone, the detection results are the best; when ResNet101 is the backbone, the detection results are very close to those when ResNet50 is the backbone. However, the amount of network parameters of ResNet101 is much larger than that of ResNet50, so we finally use ResNet50 as the backbone.

### 4.4. Study on the Image of Inspection Results

**Figure 10:**
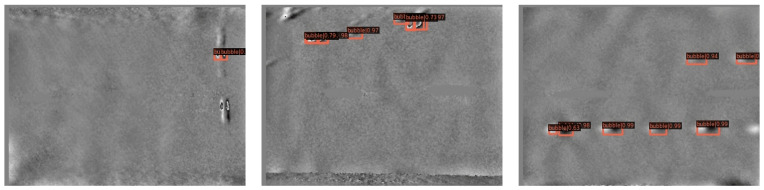
Detection results of FPN.

**Figure 11:**
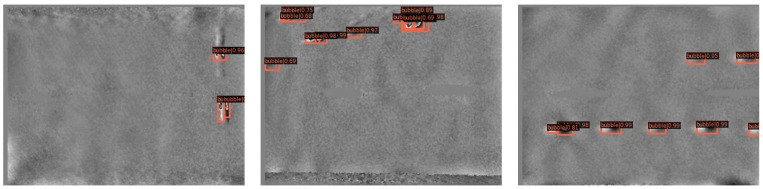
Detection results of TY-FPN.

To further show the practical effect of TYFPN, we compare the detection results and detection speed of the two methods. The detection speed is shown in Table 6. To improve the precision, TYFPN calculates more parameters in the feature fusion stage, so the detection speed of TYFPN is slightly lower than that of FPN by 12 milliseconds. In the production of tires, a CCD camera is generally used to image 12 images, and the TYFPN detection time only takes 375 milliseconds. Taking China’s national standard as an example, the automatic detection time of the tire speckle interferometry machine is less than 70 s, and our method is nearly far enough for the automatic production of tires. The detection results of Faster RCNN-FPN are shown in Figure 10, and the detection results of Faster RCNN-TYFPN are shown in Figure 11. From Figure 10 and Figure 11, it can be clearly seen that Faster RCNN-FPN has missed detection for some difficult objects, while Faster RCNN-TYFPN can detect all these difficult objects. Therefore, it can be shown that the improved network performance has improved. This means that, in the tire production process, TYFPN has higher precision and a lower missed detection rate, which can prevent defective tires from entering the market. In another sense, it can improve production efficiency, improve the automation level of the tire industry, and reduce traffic accidents caused by tire bubble defects.

## 5. Discussion

In this study, we present a neural network with a multi-directional fusion pyramid for tire defect detection. The method achieves 51.86% mAP [0.5:0.95] and 92% AP 0.5, and has better results with other neural networks with feature pyramids.

Our purpose of using a neural network is to replace manual labor and improve the precision of tire defect detection. In the previous chapter, we demonstrated that using our neural network is effective. However, neural networks are complex structures that mimic the cognitive abilities of the human brain [31], and it is not entirely clear how neural networks use the data they have been trained on to reach specific conclusions, and it is hard to determine how or why the system behaves in this way [32]. For tire defect detection, it is not only related to the interests of the industry, but also related to the safety of driving. We were unable to determine whether the neural network was biased against some types of bubble defects and chose to ignore them in detecting tire bubble defects. However, we can find and solve these problems by analyzing the results.

In the above part of the article, when we use Faster-RCNN for bubble defect detection, some bubble defects are missed, and, in the fifth set of experiments in Table 2 and Table 3, all neural networks are slightly less precise. Because some types of bubbles appear less frequently in real production and make up a small proportion of the dataset, neural networks with lower generalization ability are not sensitive to them. In the fifth set of experiments, most of these low-frequency bubble defects were divided into the test dataset, so that the neural network did not detect them well. Therefore, in order to ensure the practical application effect of neural networks in industrial defect detection, we can reduce these risks in the following ways:

1. Increase the proportion of defect types with low frequency in the dataset, increase the sensitivity of the neural network to it, and reduce the missed detection rate;
2. The equipment and environment need to be consistent, and the image sources for training and detection should be the same, such as the same camera and the same tire types, improving the detection stability of neural network;
3. Continuously tune and improve neural networks to improve precision and generalization ability.

## 6. Conclusions

In this paper, a specific and effective solution was proposed to solve the difficulties of small differences between object and background and small object size in tire crown speckle interference bubble defect detection. According to the difficulty of bubble defect detection, a multi-directional fusion feature pyramid network was proposed. In the tire bubble defect dataset, the improved network mAP [0.5:0.95] increases by 2.08%, and AP0.5 increases by 2.4%. The experimental results show that the introduction of a multi-directional fusion feature pyramid network can improve the detection performance of tire bubble defects. At the same time, it also provides a detection method and idea for rubber products with similar molding processes, such as motorcycle tires and scenes where objects are very small in object detection. In addition, with the production of tires, the tire bubble defect images continue to increase, and a complete and updated tire bubble defect database will be established in the future, and the algorithm will be continuously adjusted to train a better model.

## Figures and Tables

**Table 1:** Training parameters and details.

Optimizer	Stochastic Gradient Descent with Momentum
Batchsize	2
Max epochs	24
Environment	Ubuntu20.04 PyTorch1.6 cuda11.1
Frame	MMdetectin2.18
Equipment	GeForce RTX 3080 Intel-croe 11th i7
Dataset type	COCO Dataset

**Table 2:** mAP [0.5:0.95] evaluation metrics for different algorithms.

Algorithm	mAP [0.5:0.95]%				
	1	2	3	4	5
FasterRCNN-FPN	50.6	50.6	48.9	50.4	48.4
FasterRCNN-FPG	50.6	50.6	49.6	51.6	47.3
FasterRCNN-PAFPN	50.5	50.4	50.0	52.0	47.8
LibraRCNN(BFP)	51.2	50.6	48.8	52.4	47.9
Yolox [26]	47.4	50.3	44.3	52.0	47.8
FasterRCNN-TYFPN	**52.6**↑	**52.8**↑	**51.0**↑	**53.7**↑	**49.2**↑

**Table 3:** AP0.5 evaluation metrics for different algorithms.

Algorithm	AP0.5%				
	1	2	3	4	5
FasterRCNN-FPN	92.2	91.7	89.5	93.8	89.7
FasterRCNN-FPG	94.6	93.5	90.5	92.8	88.1
FasterCNN-PAFPN	94.9	91.4	90.8	94.8	89.4
LibraRCNN(BFP)	94.9	92.6	90.0	94.9	88.1
Yolox	89.9	91.4	86.3	94.8	89.4
FasterRCNN-TYFPN	**95.1**↑	**94.2**↑	**94.4**↑	**94.7**↑	**90.5**↑

**Table 4:** Detection results of different algorithms.

Algorithm	mAP [0.5:0.95]%	AP0.5%
FasterRCNN-FPN	49.78	91.38
FasterRCNN-FPG	49.94	91.90
FasterCNN-PAFPN	50.14	92.26
LibraRCNN(BFP)	50.18	92.10
Yolox	48.36	90.36
FasterRCNN-TYFPN	**51.86 ↑**	**93.78 ↑**

**Table 5:** Detection results of TYFPN and FPN under different backbone.

Algorithm	mAP [0.5:0.95]%	AP0.5%
RegNet50 [27] + FPN	48.84	91.20
RegNet50 + TYFPN	**49.58**	**91.36**
ResNeSt50 [28] + FPN	46.92	88.4
ResNeSt50 + TYFPN	**49.94**	**91.82**
ResNeXt50 [29] + FPN	48.9	91.46
ResNeXt50 + TYFPN	**50.8**	**92.76**
Res2Net50 [30] + FPN	49.16	91.48
Res2Net50 + TYFPN	**51.14**	**93.08**
ResNet101 + FPN	49.42	91.16
ResNet101 + TYFPN	**51.84**	**93.40**
ResNet50 + FPN	49.78	91.38
ResNet50 + TYFPN	**51.86**	**93.78**

**Table 6:** The speed of detection by different methods.

Algorithm	Speed ms/img
FPN	19.25
TYFPN	31.25

## Data Availability

Not applicable.
